# Morphological, yield, and nutritional characteristics of Chicory (*Cichorium intybus* L.) and Plantain (*Plantago lanceolata* L.) according to cutting interval in Peruvian Andes

**DOI:** 10.3389/fpls.2026.1805983

**Published:** 2026-04-24

**Authors:** Ysai Paucar, Saida Tecsi-Taipe, Luis H. Zagaceta-Llanca, Misael Rodriguez, Tony Vargas, Waldir Tarifa, Yolvi Lopez-Mendoza, José Américo Saucedo-Uriarte, Héctor V. Vásquez, Ives Yoplac, Biviana Aquino, Flor L. Mejía

**Affiliations:** 1 Instituto de Investigación en Ganadería y Biotecnología, Facultad de Ingeniería Zootecnista, Biotecnología, Agronegocios y Ciencia de Datos, Universidad Nacional Toribio Rodríguez de Mendoza de Amazonas, Chachapoyas, Peru; 2 Escuela Profesional de Ingeniería Agropecuaria, Facultad de Agronomía y Zootecnia, Universidad Nacional de San Antonio Abad del Cusco, Andahuaylas, Peru; 3 Dirección de Investigación y Desarrollo Tecnológico, Estación Experimental Agraria Andenes, Instituto Nacional de Innovación Agraria, Cusco, Peru; 4 Laboratorio de Nutrición Animal y Bromatología de Alimentos, Facultad de Ingeniería Zootecnista, Biotecnología, Agronegocios y Ciencia de Datos, Universidad Nacional Toribio Rodríguez de Mendoza de Amazonas, Chachapoyas, Peru

**Keywords:** *Cichorium intybus*, cutting interval, grasslands, high andean pastures, nutritional composition, *Plantago lanceolata*

## Abstract

In Peru, the high Andean regions made up of grasslands are important for the sustainability of local systems. Improving and promoting the sustainability of forage production and quality allows producers to be resilient in the face of constant environmental changes. In this study, the morphological characteristics (plant height, leaf length and width), yield (fresh and dry matter) and nutritional characteristics [ash, ether extract, crude protein (CP), crude fiber, neutral detergent fiber (NDF), acid detergent fiber (ADF), *in vitro* digestibility of dry matter (IVDMD), nitrogen-free extract (NFE) and gross energy] of *Cichorium intybus* L. and *Plantago lanceolata* L. were evaluated, according to the cutting interval. Six experimental plots were implemented, three per species in each of *C. intybus* and *P. lanceolata*. An analysis of variances under a linear mixed model was performed of morphological and yield characteristics, including the cutting interval (28, 35 and 42 days), and the harvest (1, 2, 3 and 4 harvests) as fixed effects, while the plants were considered as a random effect. Tukey’s multiple comparisons test (p < 0.05) was used to group the results by species using R software. The number of leaves in *C. intybus* and P. lanceolata were higher at a cutting interval of 42 days of age and in the third and fourth harvest (p<0.05). The dry matter yield of *C. intybus* and *P. lanceolata* at 42 days was higher than that of the 28 and 35 days cut, with values of 1645 kg/ha and 1545 kg/ha, respectability. The nutritional parameters of *C. intybus* and *P. lanceolata* varied significantly according to harvest age (p<0.05). Ash, CP, NDF and ADF decreased, while IVDMD and NFE increased according to age. The CP concentration was 20.84% at 28 days, 18.99% at 35 days and 17.82% at 42 days in *C. intybus* and 19.29% at 28 days, 17.80% at 35 days and 15.17% at 42 days, while the IVDMD increased from 88.5% to 94.00% in *C. intybus* and 77.82% to 88.65% in *P. lanceolata*. Harvest age is key to optimizing yield and quality of *C. intybus* and *P. lanceolata* in high Andean livestock systems.

## 1 Introduction

The grasslands of the high Andean regions of Peru are widely distributed non-forest ecosystems, with a strategic role in regulating the carbon cycle and in the sustainability of local life systems (Soto et al., 2024). These grasslands are distinguished by their heterogeneity in biomass, forage quality and floristic diversity, factors that support their productive and ecosystemic importance (Veldhuis et al., 2016). However, forage availability, especially in the mountains, is restricted by adverse climatic factors, low fertility soils and the incidence of frost, significantly limiting pasture growth throughout the year (Vallejos-Fernández et al., 2024). These restrictions directly impact local producers, whose livestock feed depends almost exclusively on grazing, affecting their productivity and food security (Merino et al., 2024a). This problem is reflected in the deterioration of the body condition of cattle and a significant reduction in their productivity, intensifying the vulnerability of high Andean production systems (Somasiri et al., 2020; Ramírez-Gómez et al., 2025). In this context, the need arises to diversify the forage base through adapted species of high nutritional value, among which the species *Cichorium intybus* L. commonly known as chicory and the species *Plantago lanceolata* L. commonly known as plantain stand out. These species are recognized for their resilience and their contribution to improving the diet of ruminants (Lee et al., 2015a; Labreveux et al., 2006; Chesney et al., 2025).

In this sense, the incorporation and management of forage species constitutes a strategic component to ensure the long-term sustainability of livestock systems. Among the most promising alternatives is *C. intybus* L (Perović et al., 2021). and the *P. lanceolata* L (Pol et al., 2021). These species have demonstrated remarkable potential as complementary forages, based on their ability to adapt to restrictive environmental conditions, their high resilience through regrowth after grazing and their superior bromatological properties, which optimize digestibility and nutritional value (Chesney et al., 2025; Labreveux et al., 2006). Results show crude protein (CP) values in chicory from 8.3 to 24.3 and for plantain 11.8 to 22.1% of CP taking into account the part of the plant, season of the year and altitude (Lee et al., 2015a; Vallejos-Fernández et al., 2024). They are now also widely used in combination with clover and kinggrass species to provide greater annual increases (Somasiri et al., 2020).

Chicory (*C. intybus* L.) is a perennial plant of the Asteraceae family, resistant to low temperatures, with branched shoots 20 to 150 cm high (Janda et al., 2021). Its chemical composition includes inulin, flavonoids, phenolic acids, sesquiterpene lactones and essential oils distributed in roots, leaves, flowers and seeds, giving it bioactive properties such as anti-inflammatory, antioxidant, hepatoprotective and antimicrobial activity (Bahmani et al., 2014). Studies have shown that the inclusion of chicory in the diet of dairy cows improves milk production and the fatty acid composition of milk, especially in the middle stages of lactation, without negatively affecting dry matter intake or energy efficiency (Mangwe et al., 2024). On the other hand, *P. lanceolata* L., an herbaceous and perennial plant of the Plantaginaceae family, has a global distribution and is widely valued for its bioactive properties (Chesney et al., 2025). Its chemical composition includes mucilages, flavonoids and iridoids, responsible for its anti-inflammatory, antimicrobial, healing and antioxidant effects (Işık and Sümer, 2025). This species is used both in the treatment of respiratory, digestive and skin conditions in humans and in veterinary medicine, as well as in the feeding of ruminants, highlighting its nutritional and functional relevance (Chesney et al., 2025). The inclusion of *P. lanceolata* L. in pastures improves milk and milk solids production, reducing urinary nitrogen excretion by 22%, with more marked effects at the end of lactation, suggesting a potential to increase productivity and reduce environmental impact in dairy systems (Nguyen et al., 2022).

Cutting age is a determining factor in the management of forage species, since it directly influences the morphology, yield and nutritional quality of the forage, that is, as the plants advance in their development, significant structural changes occur. The increase in the proportion of stems and fibers reduces digestibility, while young leaves have higher concentrations of proteins and minerals (Rupay et al., 2023). Defining an optimal cutting age allows for a balance between biomass production and nutritional quality, ensuring an adequate regrowth rate, greater pasture persistence, and efficient use of natural resources (Lemaire et al., 2009).

In this context, it is essential to evaluate how cutting interval influences the morphological characteristics, productive and nutritional performance of *C. intybus* L. and *P. lanceolata* L., key species for optimizing their integration into high Andean livestock systems. The goal is to increase feed efficiency, improve forage quality, and promote production sustainability, while strengthening producers’ resilience to agroecological constraints and promoting strategic management of high-nutritional-value forage species.

Therefore, this study aimed to determine how cutting interval affect the morphological development, forage yield, and nutritional composition of *C. intybus* L. and *P. lanceolata* L. under high-Andean conditions, to identify the most efficient management period for sustainable livestock feeding systems.

## 2 Materials and methods

### 2.1 Location of the study and soil and climate conditions

**Figure 1:**
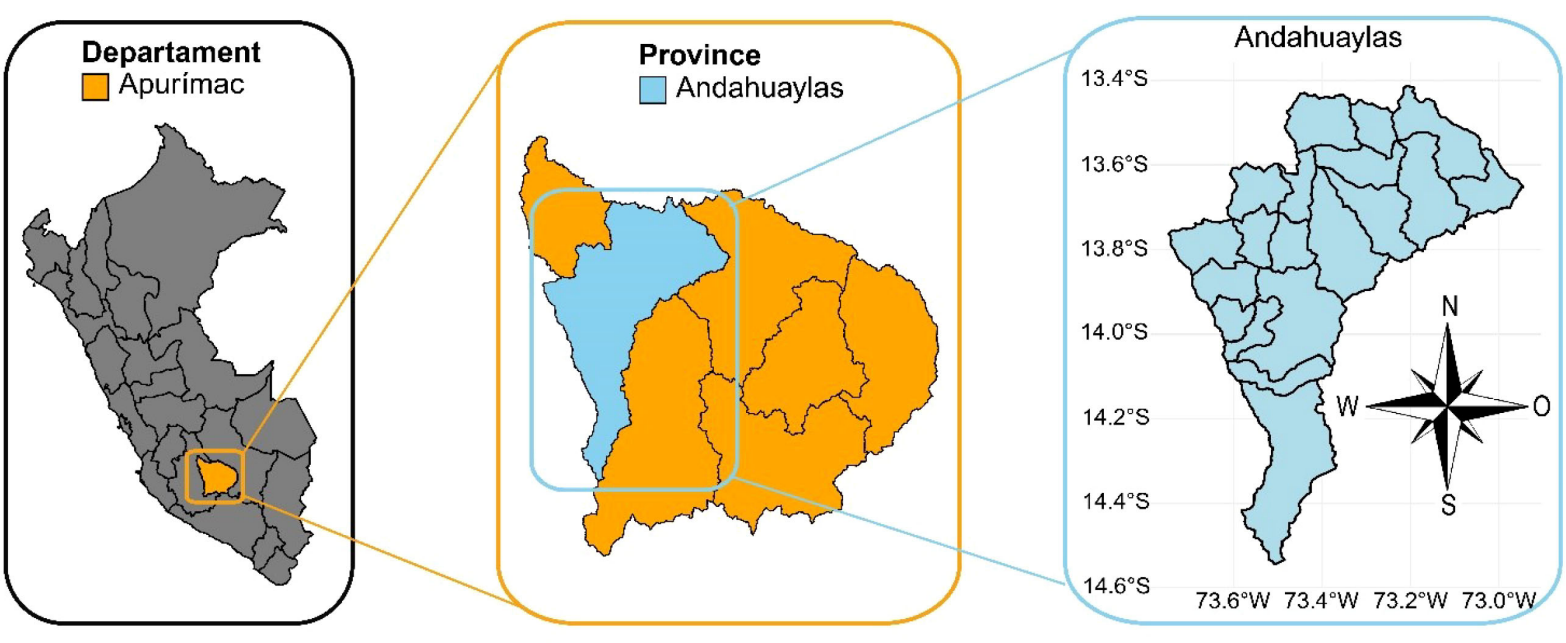
Geographic location of the experiment.

The experiment was carried out at the Choccepuquio farm of the Professional School of Agricultural Engineering of the National University of San Antonio Abad del Cusco, Peru, located at 2853 m above sea level (73°24’28.22’’ west longitude, 13°40’8.66’’ south latitude) (**Figure 1**). The area has an average annual temperature of 13.8 °C and an accumulated rainfall of 1050 mm, concentrated mainly between November and April, coinciding with the months of highest temperature; from May to October, rainfall decreases and temperatures reach their lowest values.

### 2.2 Experimental design

**Figure 2:**
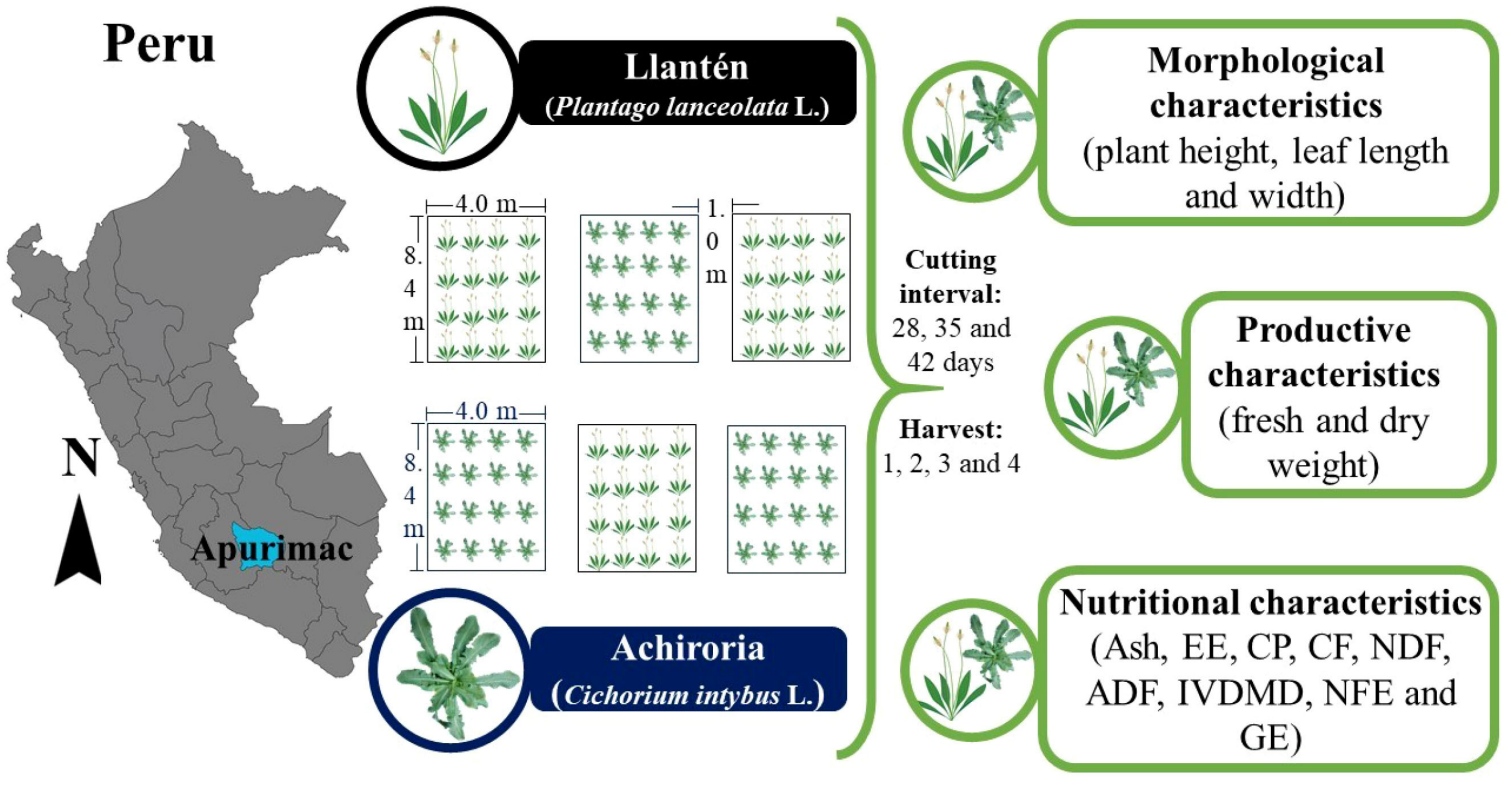
Experimental design and evaluation of variables.

A completely randomized design with three replicates was used. Six experimental plots were implemented, three per species (*C. intybus* L. and *P. lanceolata* L.), each with dimensions of 8.4 × 4.0 m and separations of 1.0 m between plots (**Figure 2**). The soil had a loamy-silty texture and homogeneous conditions, with a pH of 7.80 ± 0.00, organic matter content of 4.27% ± 0.03, total nitrogen of 0.22% ± 0.00, available phosphorus of 10.17 ppm ± 0.42 and exchangeable potassium of 291.29 ppm ± 5.75. Land preparation included mechanical leveling with a tractor and manual mulching. Prior to planting, urea was applied as a nitrogen source at a dose of 50 kg N/ha. The sowing, carried out in 2023, was carried out in a continuous jet with a spacing of 0.50 m between furrows. After 60 days, thinning was carried out to uniform plant density, establishing a final distance of 0.20 m between plants. Subsequently, after 120 days, a uniform cutting was made to a height of 4 cm.

The cultural tasks of manual weeding and sprinkler irrigation were done every 15 days, and fertilization was done at 100 kg N/ha (urea) after each cut throughout the trial. The experimental treatments corresponded to three cutting intervals: 28, 35 and 42 days. After the uniformization cut, 30 plants per species were randomly selected (10 plants per plot), which were randomly assigned to the treatments, with a distribution of 10 plants per treatment.

### 2.3 Morphological evaluation and productive yield

At each cutting interval (28, 35 and 42 days of age), morphometric variables such as plant height, leaf length and width measured with a digital vernier caliper are evaluated (Mitutoyo^®^, model 500-196-30, Japan), as well as the total number of leaves per plant. These variables were measured during four consecutive harvests. Yielding in total 30 plants (10 plants/plot x3 plots) per species. For each plant, the fresh weight were recorded using a precision balance (Ohaus, model Pioneer PA214, United States). The samples were dehydrated in a forced convection oven (Memmert^®^, model UN55, United States) at a constant temperature of 60 °C for 48 h, until constant weight is reached, in order to determine the individual dry weight (**Figure 2**). From these data, the yields of fresh and dry matter per plant (g/plant) were calculated and extrapolated to unit area (kg/ha) to estimate productivity per treatment. The dried samples were stored in airtight paper bags and kept in a controlled environment until subsequent bromatological analysis.

### 2.4 Nutritional composition

The nutritional analysis of the samples was carried out in the Laboratorio de Nutrición Animal y Bromatología de Alimentos (LABNUT) of the Universidad Nacional Toribio Rodríguez de Mendoza de Amazonas. Dry matter was determined by gravimetry (AOAC, 2023), crude protein was quantified using the Kjeldahl method 928.08 procedure using titrimetry and digestion (Sáez-Plaza et al., 2013). The total ash concentration was established by gravimetry, the ether extract was carried out through extraction with ether and subsequent gravimetry. The nitrogen-free extract was calculated by difference following the AOAC (2012). Crude fiber was analyzed using the Ankom system described by Komarek et al. (1996) and the fractions of neutral detergent fiber and acid detergent fiber were determined according to Van Soest et al. (1991). *In vitro* dry matter digestibility was estimated using the DAISY II system (ANKOM Technology, Macedon, NY, USA), according to Mabjeesh et al. (2000) and ANKOM protocol for forage. Dried and ground samples (1 mm) were weighed (0.50 g) into ANKOM F57 filter bags previously rinsed with acetone and sealed, including white correction bags. Rumen inoculum was obtained from two cattle slaughtered at the Chachapoyas Slaughterhouse. The animals came from the Molinopampa (Amazonas), whose diet was based on *Lolium multiflorum*, *Dactylis glomerata*, *Pennisetum clandestinum*, *Plantago major*, *Rumex* sp., *Holcus lanatus*, *Trifolium repens*, and *Trifolium pratense*. Rumen fluid was collected immediately after slaughter in a preheated 39 °C insulated container and transferred to the LABNUT (15 min transport time). In the LABNUT, it was homogenized using a blender (blending time: 5 seconds minimum), filtered through four layers of gauze under a continuous flow of CO_2_, and used immediately. For each digestion flask, 1600 mL of buffer solution (pH 6.8 at 39 °C) was prepared, and 400 mL of rumen inoculum was added, establishing a buffer:inoculum ratio of 4:1. Up to 25 bags per flask were incubated at 39.5 ± 0.5 °C for 48 h under constant agitation and anaerobic conditions. At the end of the incubation period, the bags were rinsed with cold water and subsequently treated with a neutral detergent solution to eliminate microbial residues, according to the NDF determination procedure. IVDMD was calculated from the blank weight loss and expressed on a dry matter basis. Finally, the gross energy was measured through bomb calorimetry (Parr 6200, United States). One gram sample was burned in an oxygen atmosphere (20–30 atm) inside a sealed bomb, measuring the temperature increase of the water in a bucket (30 °C) for 15 minutes (**Figure 2**). The choice of these parameters was based on their relevance to the comprehensive assessment of the nutritional quality of forages, as they allow for robust estimation not only of the basic chemical composition but also of the usable fraction of nutrients and their energy contribution to animal metabolism, aspects that are critical for assessing the productive efficiency and sustainability of the food systems evaluated.

### 2.5 Statistical analysis

For statistical analysis, a preliminary exploratory analysis was performed to detect outliers. Subsequently, the assumptions of normality (Kolmogorov-Smirnov) and homoscedasticity (Levene) were verified at a 95% reliability level. An analysis of variances under a linear mixed model was performed of morphological and yield characteristics, including the cutting interval (28, 35 and 42 days), and the harvest (1, 2, 3 and 4 harvests) as fixed effects, while the plants were considered as a random effect. The interaction of cutting interval and harvest was not significant, so it was not included in the models. In contrast, a one-way analysis of variance was performer for the nutritional composition, including cutting interval as factor. In case of significant differences. Tukey’s multiple comparisons test was applied (p < 0.05). All data were analyzed using the ‘agricolae’ package in R software (v.4.4.0).

## 3 Results

### 3.1 Morphological characteristics

The number of leaves was influenced by the cutting intervals in both species, showing higher values ​​on day 42 (p<0.05). Blade length and width were not affected by cutting interval in *C. intybus* L. or *P. lanceolata* L. (p>0.05), in the case of *P. lanceolata* the plant height, also was not influenced by the cutting interval. In contrast, the plant height and blade number of *C. intybus* was the affected by the cutting interval (p<0.05), showed height values at 42 days of cutting. Morphological characteristics were significantly affected by harvesting for both species (p<0.05), except for plant height and blade length in *P. lanceolata* (p>0.05). (**Table 1**).

**Table 1:** Morphological characteristics of *C. intybus* L. and *P. lanceolata* L. according to the cutting interval (Means ± SD).

Factor	n	Plant height (cm)	Blade length (cm)	Blade width (mm)	Blade number (n)
*C. intybus*					
Cutting Interval		*[0.01051]*	*[0.09553]*	*[0.1212]*	*[0.00116]*
28	40	40.6 ± 3.63ab	38.9 ± 4.16a	81.7 ± 17.6a	19.6 ± 4.9b
35	40	38.3 ± 3.27b	37.4 ± 3.39a	78.5 ± 12.7a	22.1 ± 4.1b
42	40	41.4 ± 4.12a	39.6 ± 3.9a	74.5 ± 14.4a	25.9 ± 5.99a
Harvest		*[0.00556]*	*[0.00593]*	*[<0.001]*	*[0.00375]*
1	30	42 ± 3.97a	40.6 ± 3.62a	69.5 ± 11.4b	20.9 ± 3.73c
2	30	39.4 ± 3.5b	38.1 ± 3.31b	86.8 ± 13.6a	21.3 ± 5.55cb
3	30	39.2 ± 2.83b	38.2 ± 2.78b	86.8 ± 11.2a	23.8 ± 7.47ba
4	30	39.8 ± 4.57ab	37.6 ± 5.03ab	69.9 ± 14.3b	24.2 ± 4.53a
Total	120	40.1 ± 3.89	38.6 ± 3.91	78.3 ± 15.2	22.6 ± 5.63
*P. lanceolata*					
Cutting Interval		*[0.30813]*	*[0.14602]*	*[0.319]*	*[0.03177]*
28	40	21.8 ± 3.63a	20.8 ± 3.7a	29.9 ± 9.35a	108 ± 47.3ab
35	40	22.4 ± 3.12a	21.9 ± 2.79a	32.8 ± 8.52a	78.7 ± 57.7b
42	40	23.7 ± 3.43a	23.4 ± 3.73a	34.1 ± 8.36a	125 ± 55.2a
Harvest		*[0.06875]*	*[0.07822]*	*[<0.001]*	*[<0.001]*
1	30	23.3 ± 3.52a	22.6 ± 3.96a	37.9 ± 9.08a	58.4 ± 35.5c
2	30	23.1 ± 3.46a	22.4 ± 3.73a	30.1 ± 7.71b	93.1 ± 43.8b
3	30	21.8 ± 2.8a	21.1 ± 2.89a	31.7 ± 6.36b	139 ± 53.9a
4	30	22.6 ± 3.95a	21.9 ± 3.58a	29.5 ± 9.68b	125 ± 54.9a
Total	120	22.7 ± 3.46	22 ± 3.56	32.3 ± 8.86	104 ± 56.5

Between brackets are the p-values of the analysis of variance. Different letters within columns indicate significant differences to the Tukey test (α=0.05).

### 3.2 Productive yield in *C. intybus* L. y *P. lanceolata* L.

**Figure 3:**
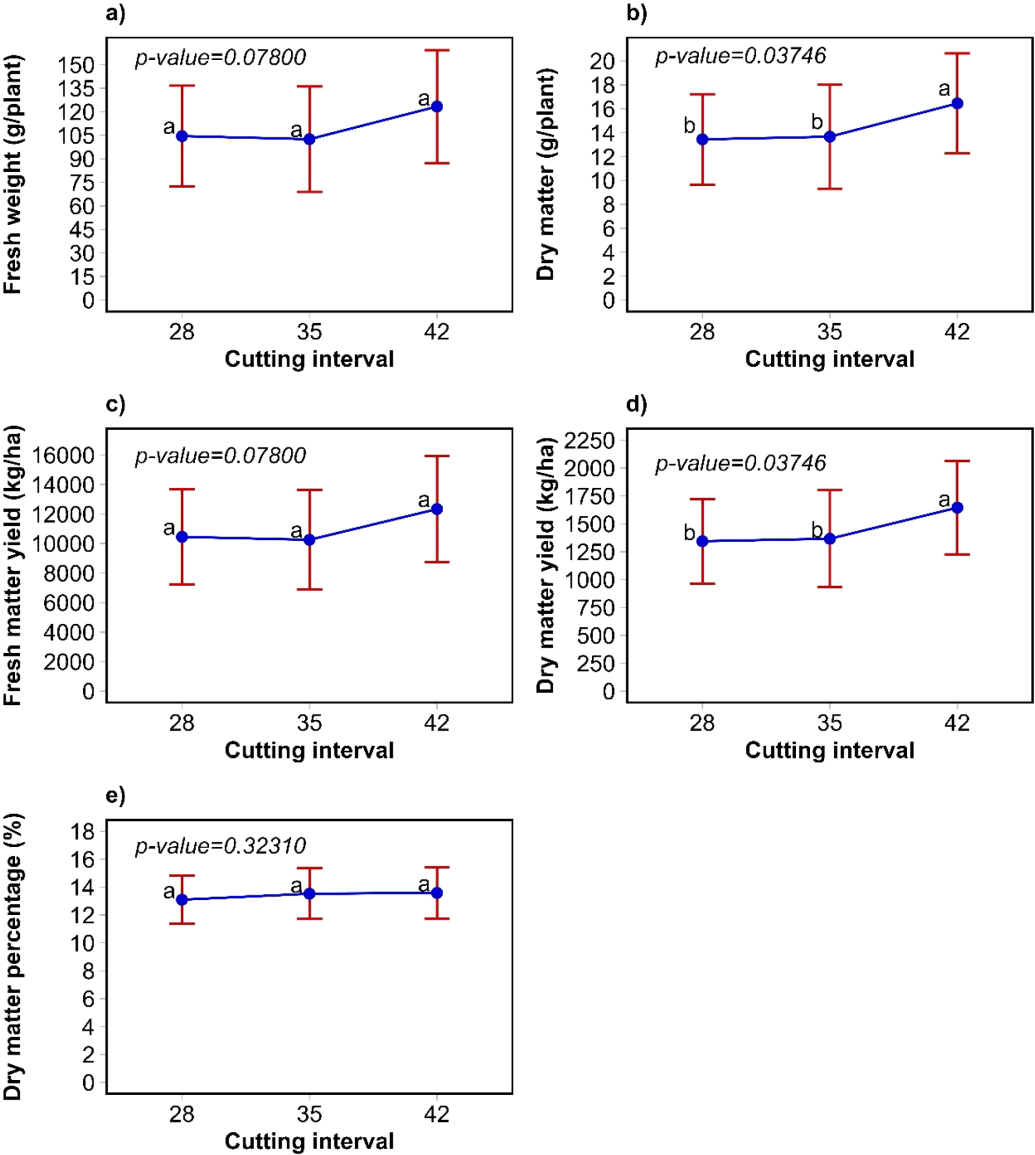
Weight of individual plants **(a, b)**, yield **(c, d)**, and dry matter percentage **(e)** of *C. intybus* according to cutting interval. The bars are the standard deviation, different letter in indicate significant differences to the Tukey test (α=0.05).

The dry matter yield for *C. intybus*, at cutting intervals of 28, 35 and 42 days were 1344, 1367 and 1645 kg/ha, respectively. According to the cutting interval, the highest dry matter production was achieved at 42 days for *C. intybus* (p<0.05; **Figures 3b, d**). In contrast, the fresh weight of individual plants, as well as their yield and dry matter percentage, were not affected by the cutting interval (p>0.05, **Figures 3a, c, e**). The annual dry matter yield (kg/ha/year) for *C. intybus* were 17472, 14217 and 14312 for cutting intervals of 28, 35 and 42 days, respectively.

**Figure 4:**
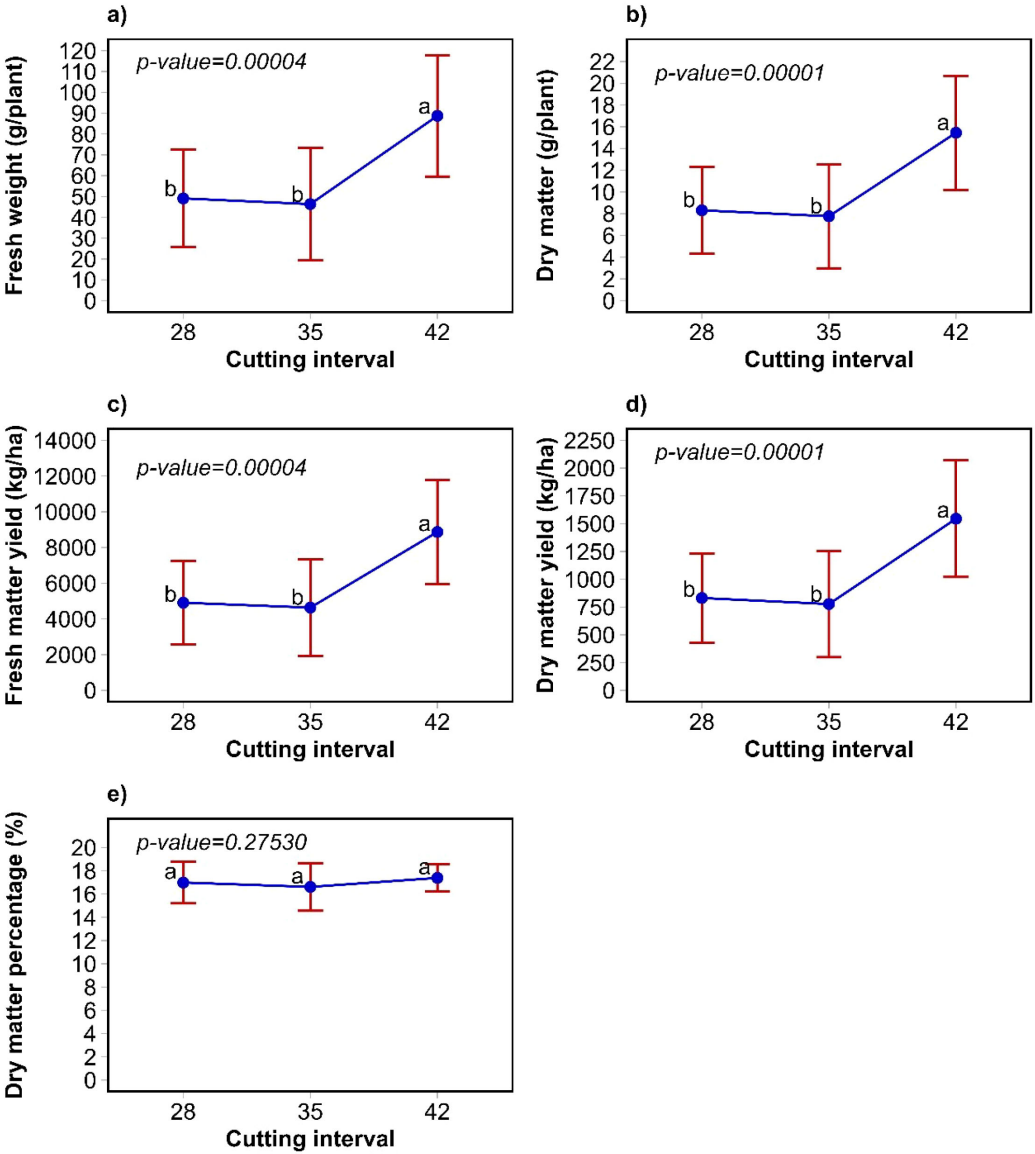
Weight of individual plants **(a, b)**, yield **(c, d)**, and dry matter percentage **(e)** of *P. lanceolata* according to cutting interval. The bars are the standard deviation, different letter in indicate significant differences to the Tukey test (α=0.05).

The dry matter yield for *P. lanceolata*, at cutting intervals of 28, 35 and 42 days were 831, 777 and 1545 kg/ha, respectively. According to the cutting interval, the highest fresh and dry matter production were achieved at 42 days for P. lanceolata (p<0.05; **Figures 4a–d**). On the other hand, the dry matter percentage was not affected by the cutting interval (p>0.05, **Figure 4e**). The annual dry matter yield (kg/ha/year) for *P. lanceolata* were 10803, 8081 and 13442 for cutting intervals of 28, 35 and 42 days, respectively.

In *C. intybus*, fresh weight per plant and fresh matter yield were higher in the third harvest (p<0.05). However, no differences were observed in dry matter per plant and dry matter yield (p>0.05). The percentage of dry matter was higher in the fourth harvest (p<0.05). For *P. lanceolata*, the harvest influenced individual plant weight and yield, as well as dry and fresh matter, showing higher values ​​from the third harvest onward (**Table 2**).

**Table 2:** Means (standard deviation) of plant weight of individual plants, yield and dry matter percentage of *C. intybus* and *P. lanceolata* according to the harvest.

Factor	N	Fresh weight (g/plant)	Dry matter (g/plant)	Dry matter percentage (%)	Fresh weight yield (kg/ha)	Dry matter yield (kg/ha)
*C. intybus*						
Harvest		*[0.00545]*	*[0.09637]*	*[<0.001]*	*[0.00545]*	*[0.09637]*
1	30	112 ± 28.4ab	13.8 ± 3.16a	12.4 ± 0.81c	11217 ± 2839ab	1377 ± 316a
2	30	99.5 ± 24.5b	13.6 ± 3.28a	13.7 ± 0.829b	9952 ± 2451b	1364 ± 328a
3	30	125 ± 36.2a	15.3 ± 4.77a	12.2 ± 1.16c	12499 ± 3616a	1528 ± 477a
4	30	104 ± 43.7b	15.4 ± 5.45a	15.3 ± 1.96a	10387 ± 4366b	1540 ± 545a
Total	120	110 ± 34.9	14.5 ± 4.3	13.4 ± 1.79	11014 ± 3494	1452 ± 430
*P. lanceolata*						
Harvest		*[<0.001]*	*[<0.001]*	*[<0.001]*	*[<0.001]*	*[<0.001]*
1	30	42.4 ± 24.6c	6.77 ± 3.99c	15.9 ± 1.31b	4240 ± 2465c	677 ± 399c
2	30	54.9 ± 28.4b	9.54 ± 4.78b	17.5 ± 1.66a	5491 ± 2845b	954 ± 478b
3	30	73.1 ± 31.8a	12.2 ± 5.99a	16.3 ± 1.83b	7307 ± 3176a	1221 ± 599a
4	30	75.1 ± 35a	13.5 ± 6.13a	18.2 ± 1.02a	7511 ± 3501a	1352 ± 613a
Total	120	61.4 ± 32.8	10.5 ± 5.84	17 ± 1.73	6137 ± 3276	1051 ± 584

Between brackets are the p-values of the analysis of variance. Different letters within columns indicate significant differences to the Tukey test (α=0.05).

### 3.3 Nutritional composition

The nutritional parameters evaluated, including percentage of dry matter, mineral content, crude protein, neutral detergent fiber (NDF), acid detergent fiber (ADF), *in vitro* digestibility of dry matter, nitrogen-free extract, and gross energy, showed significant variations depending on the days of cutting in both *C. intybus* L. and *P. lanceolata* L. (p < 0.05). The concentration of the ether extract showed no differences in *C. intybus* L. (p > 0.05), while in *P. lanceolata* L. a significant increase was observed at 42 days, only in comparison with the cut made at 28 days. On the other hand, crude fiber did not show significant changes in *P. lanceolata* L. (p > 0.05); however, in *C. intybus* L. a decreasing trend was evident as the cutting interval increased (**Table 3**).

**Table 3:** Nutritional characteristics of *C. intybus* and *P. lanceolata* according to the cutting interval (Means ± SE).

Cutting interval	DMP (%)	Ash (%)	EE (%)	CP (%)	CF (%)	NDF (%)	ADF (%)	IVDMD (%)	NFE (%)	GE (kcal/kg)
*C. intybus*										
p-valor*	<0.050	<0.050	0.542	<0.050	<0.050	<0.050	<0.050	0.029	<0.050	0.042
28 days	11.14± 0.03b	11.88 ± 0.22a	2.45 ± 0.06a	20.84 ± 0.20a	18.05 ± 0.18a	22.92 ± 0.12a	19.44 ± 0.06a	89.78 ± 1.10ab	39.98 ± 0.33b	3834.61 ± 21.33a
35 days	11.19 ± 0.11b	11.11 ± 0.11b	2.54 ± 0.12a	18.99 ± 0.34b	14.52 ± 0.38b	21.63 ± 0.56a	17.86 ± 0.39b	88.5 ± 1.39b	45.55 ± 1.04a	3824.75 ± 32.33a
42 days	11.89 ± 0.02a	9.66 ± 0.18c	2.6 ± 0.08a	17.82 ± 0.32c	11.87 ± 0.50c	16.91 ± 0.38b	13.72 ± 0.21c	94 ± 1.22a	47.75 ± 0.92a	3746.11 ± 4.97a
Total	11.4 ± 0.11	10.88 ± 0.29	2.53 ± 0.05	19.21 ± 0.40	14.81 ± 0.79	20.49 ± 0.81	17.01 ± 0.74	90.76 ± 0.96	44.43± 1.08	3801.82 ± 16.77
*P. lanceolata*										
p-valor*	<0.050	<0.050	<0.050	<0.050	0.263	<0.050	<0.050	<0.050	<0.050	<0.050
28 days	16.07 ± 0.11a	12.02 ± 0.41a	1.84 ± 0.11b	19.29 ± 0.26a	11.39 ± 0.47a	33.4 ± 0.37a	25.11 ± 0.11a	77.82 ± 0.69b	48.36 ± 0.86b	3762.21 ± 28.14b
35 days	14.65 ± 0.01c	11.00 ± 0.13a	2.13 ± 0.06ab	17.80 ± 0.19b	10.66 ± 0.50a	26.41 ± 0.18b	18.78 ± 0.37b	81.44 ± 1.09b	53.12 ± 0.71a	3875.80 ± 8.48a
42 days	15.62 ± 0.01b	9.51 ± 0.18b	2.55 ± 0.16a	15.17 ± 0.25c	10.27 ± 0.37a	20.29 ± 0.46c	14.28 ± 0.52c	88.65 ± 1.11a	52.85 ± 0.67a	3740.43 ± 8.12b
Total	15.45 ± 0.18	10.84 ± 0.34	2.17 ± 0.11	17.42 ± 0.53	10.77 ± 0.28	26.70 ± 1.62	19.39 ± 1.35	82.63 ± 1.45	51.44 ± 0.77	3792.81 ± 20.12

*, p-value that evaluate de effect of the populations; abc, different letters in each column show significant differences to the Tukey test (α = 0.05). DMP, EE, CP, CF, NDF, ADF, IVDMD, NFE and GE) ash, EE, ether extract; CP, crude protein; CF, crude fiber; NDF, neutral detergent fiber; ADF, acid detergent fiber; IVDMD, *in vitro* digestibility of dry matter; NFE, nitrogen-free extract; GE, gross energy; (n =4); SE, standard error.

In both species, a progressive decrease in mineral content, crude protein, neutral detergent fiber, and acid detergent fiber was observed as the cutting interval increased, while nitrogen-free extract and *in vitro* dry matter digestibility showed an opposite trend, increasing as the vegetative cycle progressed. In the case of *C. intybus* L., the percentage of dry matter increased consistently with the number of days since cutting, reflecting a greater accumulation of structural biomass, while in *P. lanceolata* L. an inverse behavior was evident, with a reduction in dry matter content as the harvest interval was prolonged, suggesting physiological differences between species that condition their productive response and their nutritional value.

## 4 Discussion

The present study, carried out under representative high Andean conditions, confirmed that the cutting interval is a key management tool to balance the production and nutritional quality of *C. intybus* L. and *P. lanceolata* L.

### 4.1 Morphological characteristics

The results show how the cutting interval and harvests modify height, blade length and width, and blade number of *C. intybus* and *P. lanceolata* (**Table 1**), showing an increase in the blade number at a 42-day interval, without changes in blade height or blade length. These results are consistent with studies demonstrating that longer regrowth intervals allow for greater accumulation of leaf structures and increase in total biomass (Merino et al., 2024a; Mangwe et al., 2020; Lee et al., 2015a). These results highlight the importance of adjusting the cutting interval and harvest according to the species to optimize both the productivity and persistence of the grass in sustainable forage systems.

In *P. lanceolata*, the greater number of leaves is consistent with the plant’s rosette-shaped and branched architecture, superficial root system and smaller plants compared to *C. intybus*, a taller plant with a deep taproot (Cranston et al., 2016; Sanderson and Elwinger, 2000). Under water stress, *C. intybus* invests more in deep roots and shows greater persistence, while *P. lanceolata* maintains a higher fraction of aerial biomass and can be more productive in moderate drought (Cranston et al., 2016).

The blade number increases in both species in successive harvests. This is because the plants are producing more biomass. In the *C. intybus* and *Pennisetum purpureum* cv. Mott association, plant height could be associated with greater biomass production at different cutting intervals (Zaini et al., 2021).

### 4.2 Productive yield in *C. intybus* L. y *P. lanceolata* L.

The results for *C. intybus* showed that harvests significantly influenced fresh weight per plant and fresh weight yield per hectare, while dry matter per plant and dry matter yield did not show significant differences between harvests (**Table 2**). The highest fresh weight per plant and forage yield were recorded in the third harvest, reaching values ​​of 125 g/plant and 12499 kg/ha, respectively. This pattern has been observed in other studies indicating that intermediate regrowth tends to have higher biomass production than the first cut, due to the prior establishment of the crop and the development of reserves in the taproot (Zaini et al., 2021). The fresh forage yield was higher than that reported by Montalvo (2018), who found 3364.17 kg/ha of yield at 170 days, based on a single cut on broadcast sowing system, using fruit tea-based foliar fertilizer. This difference can be attributed to the cutting interval, which was 170 days, without a prior homogenization cut, under a broadcast sowing system. Although the yield (kg/ha/cut) was higher at 42 days, the annual yields of *C. intybus* are highest at 28 days (17472 kg/ha/year), more than 3.1 tons higher than the other cutting intervals. These results are higher than the 14588 kg/ha/year reported by Sanderson (2007) in three varieties of chicory cut at 30 days. These results highlight that a higher annual yield would be obtained at 28 days of cutting, this constitutes important information for decision-making in the management of this species.

The percentage of dry matter in *C. intybus* was similar in the cutting interval and harvest, with the highest value (15.3%) recorded in the fourth harvest. These results may be related to greater physiological maturity of plant tissues and increased biomass accumulation, which occurs with increasing age. In *C. intybus*, increasing harvest age leads to greater aboveground biomass accumulation and changes in plant tissue composition, resulting in increased dry matter. Furthermore, the growth and regrowth dynamics of *C. intybus* are influenced by management factors such as defoliation frequency, nutrient availability, and the plant’s ability to mobilize reserves from the root, which can generate variations in production between different harvest cycles (Zaini et al., 2021). This confirms that these species can maintain a relatively stable dry matter production over several harvest cycles.

In *P. lanceolata*, the harvests and the cutting interval had significant effect on the evaluated variables, except for dry matter percentage cording to cutting interval. The fresh weight per plant showed a progressive increase from the first harvest (42.4 g/plant) to the fourth (75.1 g/plant), reaching the highest values ​​in harvests 3 and 4. This behavior can be attributed to the ability of this species to develop a deep root system and rapid leaf expansion, which favors biomass accumulation and regrowth after defoliation, guaranteeing high levels of production throughout successive cuts (Vallejos-Fernández et al., 2024). Similarly, fresh weight yield per hectare showed significant differences between harvests, increasing in consecutive harvests. This increase reflects *P. lanceolata* ability to accumulate biomass over time, especially when the plants reach greater physiological development, highlighting its productive potential compared to other herbaceous species used in high-Andean pastures (Vallejos-Fernández et al., 2024). The 42-day cutting interval showed the highest value ​​for fresh weight per plant, dry matter per plant, fresh weight yield, and dry matter yield of *P. lanceolata*, exhibiting significant differences compared to the shorter intervals. This behavior is explained by the fact that a longer interval between cuttings allows plants more time for leaf expansion, reserve accumulation, and recovery after defoliation, which increases total biomass production. Recent modeling of *P. lanceolata* growth and defoliation also indicates that longer defoliation intervals favor greater biomass accumulation and forage yield (Cichota and Yang, 2025). The annual dry matter yield was 13442 kg/ha/year at 42 days, followed by 10803 kg/ha/year at 28 days and 8081 kg/ha/year at 35 days, demonstrating superiority at 42 days of cutting. Under the conditions of the Peruvian Andes, an annual yield of 13390 kg/ha/year was reported for *P. lanceolata* with seven annual harvests (Vallejos-Fernández et al., 2024). To obtain higher yields in this species, it should be harvested at longer cutting intervals. These forage species are preferred by sheep and lambs, with *C. intybus* L. consumption being approximately twice that of ryegrass, leading to a decrease in methane production (Niderkorn et al., 2019; Somasiri et al., 2020). Furthermore, mixed pastures of *C. intybus* L. and *P. lanceolata* L. contribute to increased milk production, which translates into greater weight gain in lambs (Kenyon et al., 2017; Rodríguez et al., 2020; Kemp et al., 2010). Implementing differentiated cutting intervals can enhance forage utilization efficiency, high-quality biomass, may help farmers increase animal productivity, reduce environmental impacts and promote the long-term sustainability of grazing systems.

### 4.3 Nutritional composition

In *P. lanceolata* L., increasing the cutting interval has been shown to result in a progressive decrease in crude protein (CP), minerals and fiber (NDF and ADF), while *in vitro* digestibility and soluble extracts (NFE) increased significantly. Furthermore, an increase in dry matter content was observed when the cutting interval was longer *in C. intybus* L. while a slight reduction was observed in *P. lanceolata* L. This was also observed in dry matter digestibility (**Table 2**). These results suggest that each species regulates the relationship between structural accumulation and soluble fractions differently, which could significantly influence feed conversion ratios (Merino et al., 2024b).

In our study, CP decreased with the cutting interval. This behavior was also observed by Merino et al. (2024a), who demonstrated a decrease in protein content at higher cutting heights in *P. lanceolata*. In *C. intybus*, decrease CP protein content was observed depending on the time of evaluation, with a higher concentration in the early periods (Ma et al., 2026).

The *in vitro* dry matter digestibility of *C. intybus* was higher than that of *P. lanceolata* and increased with cutting intervals, reaching values ​​of 90.76% and 88.65%, respectively at 42 days. This behavior may be related to changes in the cellular composition of the forage tissue (lower NDF and ADF) and the accumulation of structural compounds that are more easily degradable in the rumen. These results are consistent with previous studies that report digestibility’s of 93.2% in puna chicory and values ​​of 87 in *P. lanceolata*, which indicates an improvement in the availability of digestible nutrients and highlights its potential as a supplement in ruminant diets (Labreveux et al., 2006). Furthermore, Vallejos-Fernández et al., 2024, report higher values in the two varieties of chicory compared to plantain in different altitudinal levels; however, these values were lower than the results of this research, thus reinforcing the importance of these species to adapt to different altitudinal levels. These findings suggest that *C. intybus* L. has a higher efficiency in converting dry matter into usable energy for animals. Furthermore, proper grazing management and cutting height are essential to optimize the nutritional quality of these forage species (Labreveux et al., 2006; Sanderson et al., 2003).

Our results show a significant reduction in fiber values, particularly NDF and ADF, as the cutting interval increases. That are supported by those found by Ma et al. (2026), who found no differences for ADF of leaves among three evaluation periods (nutritional period, flowering period and fruiting period). However, they found differences for NDF, but the flowering and fruiting period showed similar values (Ma et al., 2026). While the general trend indicates that NDF and ADF increase with maturity due to greater lignification and cell wall development, this response is not universal. In our study, the observed decrease can be explained by changes in the leaf:stem ratio, as described by Lee et al. (2015a), who found that as defoliation intervals increase, the yield of new leaves also increases. In our study the blade number increased with the cutting interval for chicory and plantain, this species doesn’t have stems compared with other traditional pastures, as grasses and legumes. The environment can also affect fiber behavior, in the Peruvian Andes the NDF decreases at higher altitudes (Vallejos-Fernández et al., 2024). Moderate temperatures and radiation patterns diminish lignification processes compared to temperate or lowland systems, modifying the typical fiber accumulation pattern reported in other environments. For example, in conditions of Larson Agricultural Research Center, Sanderson et al. (2003) reported NDF levels of 435 g/kg in puna chicory and 502 g/kg for plantain. In our study the NDF were 169.1 g/kg and 202.9 g/kg at 42 days for *C. intybus* L. and *P. lanceolata* L., respectively. Indicating comparatively lower fiber concentrations under our experimental conditions.

It has been shown that, with 42 days of cutting interval, the *C. intybus* L. and P. Lanceolata maximize their biomass production. It has been demonstrated that these species reach adequate reserves of non-structural carbohydrate at 21 days (Lee et al., 2015b). Furthermore, Studies conducted by Belesky et al. (2000) reinforce the need to use chicory in forage mixtures to meet energy concentrations, and one of these forages is plantain in monocultures (Belesky, 1999). This complementarity allows for alternating cuts and combining plantings, optimizing both the quality and availability of forage and enhancing the benefits of each species in mixed production systems. This approach is relevant in high Andean systems, where soil and climatic conditions demand efficient and resilient alternatives to ensure a sustainable forage supply (Guo et al., 2025; Lorenz et al., 2020). Implementing mixed pastures of chicory and plantain under 42-day cutting intervals al the first year, could improve forage production, increase dietary energy supply for livestock, and contribute to the long-term sustainability of Andean grazing systems. Biomass production in the first year of perennial species is key to guarantee stable yield (Cooney et al., 2023), and future studies should consider long-term assessment to evaluate their biomass yields and species persistence.

The bromatological analyses carried out in the studies clearly show the differences that exist between both species through the days on which each nutritional analysis was performed (Jaramillo et al., 2021; Vallejos-Fernández et al., 2024). This knowledge allows producers to select appropriate cutting intervals and grazing strategies that maximize forage quality and animal performance while maintaining pasture persistence and soil health.

### 4.4 Limitation

This study shows important results regarding morphological characteristics, yield, and nutritional composition of C. intybus and P. lanceolata, according to cutting intervals, during four consecutive harvests. However, the following limitations persist: The study was limited to four consecutive harvests during the first year, without considering economic factors, and focused on manual cutting, not direct grazing by animals. These aspects should be considered for future research.

## 5 Conclusion

*C. intybus* and *P. lanceolata* increase the number of leaves with significant changes according to cutting interval, in *C. intybus* the plant height was also influenced by the cutting interval. The harvests have influenced in all morphological characteristics of *C. intybus* and *P. lanceolata*, except for plant height and blade length of *P. lanceolata.* The dry matter yield of both species at 42 days was superior to that of the 28 and 35-day cuts, demonstrating that harvest frequency influenced forage production, with the exception of the annual yield observed in *C. intybus*. The annual dry matter yield of *C. intybus* is higher under a 28-day cutting interval, whereas plantain reaches its maximum annual dry matter production potential at 42 days. Nutritionally, *C. intybus* is distinguished by its higher digestibility and crude protein content, while *P. lanceolata* maintains stability in its components. These results confirm that managing the cutting interval during the first year of growth is key to optimizing agronomic, productive, and nutritional characteristics. Future research should include long-term studies to confirm the productive potential and persistence of the species in high Andean zones.

## Data Availability

The data supporting the findings of this study are available from the corresponding author upon reasonable request.
